# Renin-angiotensin system inhibitor use and risk of Parkinson’s disease: a meta-analysis

**DOI:** 10.1007/s13760-024-02560-7

**Published:** 2024-04-26

**Authors:** Tian-xiang Xu, Hai-yin Jiang, Zeng-yan Yang

**Affiliations:** 1 Guangxi University of Chinese Medicine, Nanning, Guangxi China; 2 State Key Laboratory for Diagnosis and Treatment of Infectious Diseases, Collaborative Innovation Center for Diagnosis and Treatment of Infectious Diseases, The First Affiliated Hospital, College of Medicine, Zhejiang University, Hangzhou, China

**Keywords:** Antihypertensive medication, Hypertension, Move disorder, Neurodegenerative

## Abstract

**Background:**

Hypertension is a recognized risk factor for Parkinson’s disease (PD). The renin-angiotensin system (RAS) inhibitors are widely used to treat hypertension. However, the association of RAS inhibitor use with PD has still been an area of controversy.

**Methods:**

Thus, we conducted a meta-analysis to investigate the relationship between RAS inhibitor use and PD. PUBMED and EMBASE databases were searched for articles published up to Oct 2023. All studies that examined the relationship between RAS inhibitor use and the incidence of PD were included.

**Results:**

Seven studies with total 3,495,218 individuals met our inclusion criteria for this meta-analysis. Overall, RAS inhibitor use was associated with a reduction in PD risk (OR = 0.88, 95%CI = 0.79–0.98) compared with the controls. When restricted the analysis to individuals with RAS inhibitor use indication, RAS inhibitor exposure was also associated with a decreased risk of PD (OR = 0.76, 95%CI = 0.62–0.92). Pooled results of cohort studies also did support a protective role of angiotensin converting enzyme inhibitors (ACEIs) (OR = 0.97, 95%CI = 0.89–1.07) users and angiotensin II receptor blockers (ARBs) (OR = 0.8, 95%CI = 0.63–1.02) in PD.

**Conclusion:**

Overall, RAS inhibitor use as a class is associated with a reduction in PD risk. However, the findings of ACEIs and ARBs may be limited by small sample size. Future well-designed studies considering the classification by inhibitor type, duration, dose, or property of BBB penetration of RAS inhibitors are needed to clarify the contribution of these exposure parameters on the risk of PD.

**Supplementary Information:**

The online version contains supplementary material available at 10.1007/s13760-024-02560-7.

## Introduction

Parkinson’s disease (PD) is a chronic, progressive neurodegenerative disorder characterized by symptoms such as tremor, muscle rigidity, bradykinesia, and postural instability [1]. It is the predominant movement disorder among the elderly, affecting 1% of individuals  > 60 years of age [2]. The aging of the worldwide population suggests that the PS incidence may nearly double by 2050 [3]. PD significantly diminishes quality of life and imposes a considerable economic burden on health systems, highlighting the need to explore potential risk factors and protective agents against this disease.

The renin-angiotensin system (RAS) is an essential circulating hormonal system that is instrumental in regulating blood volume, electrolyte balance, and systemic vascular resistance [4]. Angiotensin II (Ang II), the main active component of RAS, mediates its effects primarily through two receptor subtypes, AT1 and AT2, which are widely expressed in a variety of tissues including in the brain [5]. Recent preclinical studies have provided evidence that RAS inhibition may reduce dopaminergic neuronal loss and mitigate oxidative stress in animal models of PD by inhibiting mitochondrial function [6, 7]. However, the results of animal studies are not always generalizable to human clinical settings. Initial studies did not demonstrate an association of the use of angiotensin-converting enzyme inhibitors (ACEIs) and angiotensin II receptor blockers (ARBs) with the risk of PD in the general population [8–10]. Nonetheless, recent findings from a study suggest that the use of ARBs may be linked with a decreased risk of PD in hypertensive individuals [11]. Similarly, a substantial Norwegian cohort study by Romanowska et al. [12] found an association between RAS medications and a reduced risk of PD in the general population. Moreover, two other cohort studies identified a lower PD risk in patients treated with ACEIs [13, 14]. The relationship between RAS inhibitors and PD risk is further complicated by hypertension, which not only is the primary condition for which RAS inhibitors are prescribed but also has been associated with an increased risk of PD, introducing potential confounding by indication.

Given the contradictory evidence, the connection between RAS inhibitors and PD risk remains controversial. Considering the prevalent use of RAS inhibitors, it is crucial to understand their long-term impact on PD, particularly their influence on disease incidence. Consequently, this meta-analysis systematically reviewed the literature to clarify the association between RAS inhibitor use and subsequent PD risk.

## Methods

### Search strategy

The present systematic review was conducted in compliance with the PRISMA (Preferred Reporting Items for Systematic Reviews and Meta-Analyses) statement. A comprehensive literature search of the PubMed and Embase databases was performed to identify relevant articles from database inception to Oct 1, 2023. The search strategies were using the following search terms and their variants individually or in combinations: “renin-angiotensin aldosterone system OR renin–angiotensin–aldosterone system AND renin-angiotensin system OR renin angiotensin system OR RAAS OR RAASi OR RAS OR angiotensin converting enzyme inhibitors OR ACE inhibitors OR angiotensin II receptor blockers OR ARBs OR ARB” and “Parkinson OR Parkinsonism OR Lewy Body”. The reference lists of the retrieved publications were manually searched to identify additional studies.

### Inclusion criteria

Peer-reviewed studies published in English were considered eligible if they met the following PICOS criteria: (1) population: adults with or without an indication for anti-hypertensive treatment; (2) exposure: renin-angiotensin system inhibitors use (ACEIs or ARBs); (3) comparison: no treatment or other anti-hypertensive treatment; (4) primary outcome: incidence of PD; and (5) study design: randomized-controlled trials, case-crossover or self-controlled case series, and cohort, nested case–control, and case–control studies.

### Data extraction

Extracted data included authors, year of publication, location, study design, number of participants, inclusion/exclusion criteria, details of renin-angiotensin system inhibitors use (timing and indication of use and type), details of comparison (no anti-hypertensive or other anti-hypertensive treatment), outcomes and their assessment methods and definitions, statistical adjustment for confounders, and quality. Adjusted estimates with their variances were extracted when available; when adjusted estimates were not provided in the published data, we calculated crude odds ratios (OR).

### Quality assessment

Each manuscript included was independently assessed using the Newcastle–Ottawa Quality Assessment Scale (NOS) [15], as recommended by the Cochrane Collaboration for evaluation of observational study quality. A score of  > 7 points was considered an indicator of a high-quality study. This scale rates the quality of an observational study in three aspects: selection (four questions) and comparability (two questions) of the study groups, along with the exposure (case–control) or outcome (cohort) of interest (three questions); all questions are scored 0 or 1.

### Outcome assessment

The primary outcome assessed was the risk of PD with renin-angiotensin system inhibitors use compared with no renin-angiotensin system inhibitors use. Subgroup analyses were conducted according to study design (case–control or cohort) and classification of renin-angiotensin system inhibitors (ACEI or ARB). We further conducted analysis limited to studies controlled the drug indication.

### Statistical analysis

The analyses were performed using Stata 12.0 software (Stata Corp., College Station, TX, USA). The heterogeneity level was assessed using the *I*^*2*^ statistic; *I*^*2*^ > 50% were considered to indicate substantial heterogeneity [16]. The risks of PD are expressed as the OR with the 95% confidence intervals (CI) for case–control studies and RR or HR with the 95% CI for cohort studies. ORs were considered approximations of RRs or HRs because the outcome under study is rare in all populations. Therefore, we employed the OR parameter with a 95% CI using a DerSimonian and Laird method in view of the clinical heterogeneity among the included studies [17]. Finally, funnel plots and Egger's test were used to evaluate the presence of publication bias within this meta-analysis [18, 19]. Statistical significance was set at a *P* < 0.05.

## Results

### Search results

**Fig. 1:**
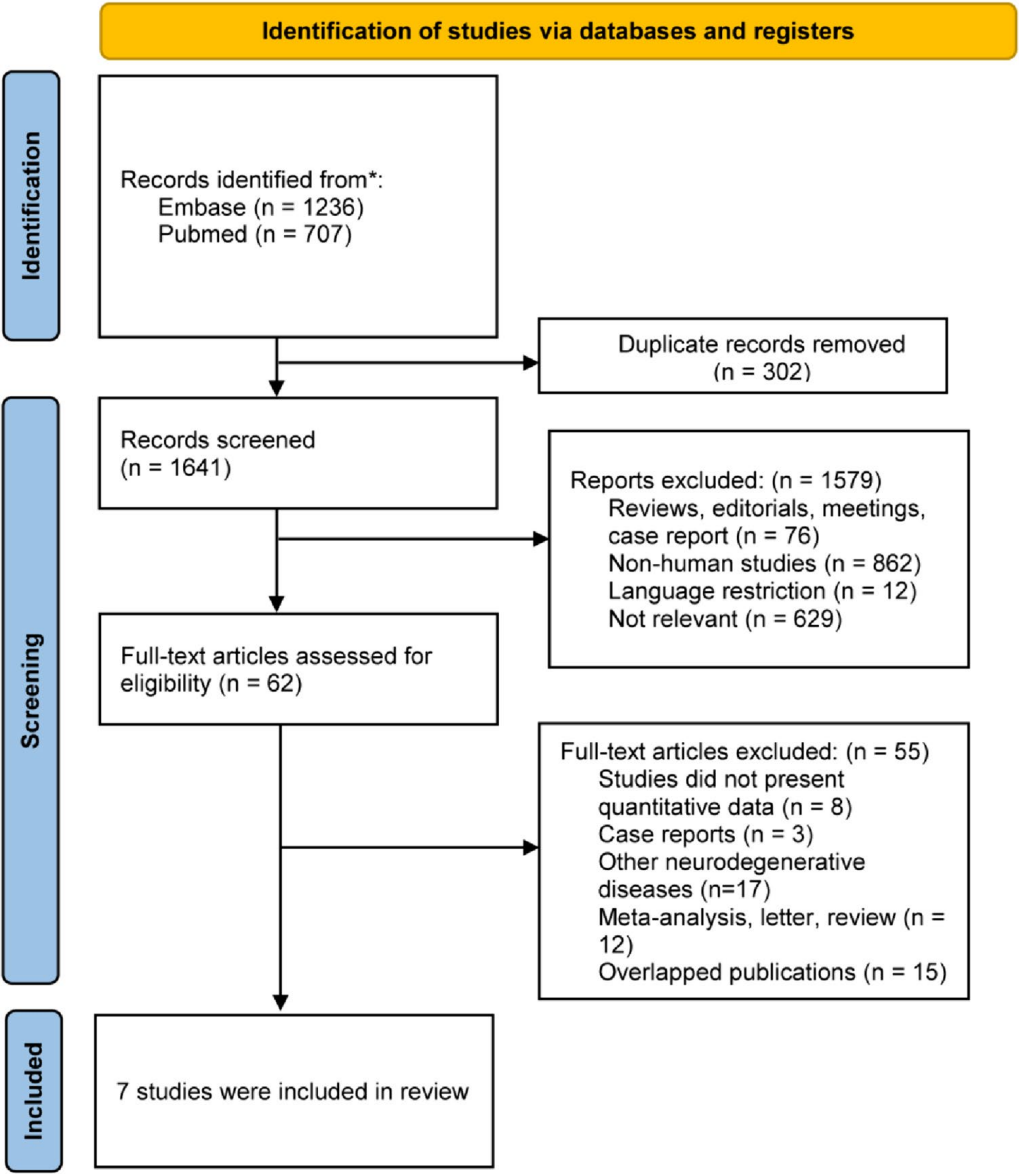
Flowchart of the studies considered and finally selected for review

The study selection process is depicted in Fig. 1. A total of 1641 citations were identified across two databases after the removal of 302 duplicates. Following the screening of titles and abstracts, 1579 studies were excluded, leaving 62 for full-text review. Of these studies, 55 were excluded due to various reasons. Ultimately, seven studies [8–14] encompassing 3,495,218 individuals were included in the meta-analysis.

### Characteristics of included studies

Table 1 details the main characteristics of the included studies, which all used a population-based observational design (three case–control and four cohort studies). These studies were published between 2008 and 2023, with sample sizes ranging from 7274 to 3,223,637 participants. Four studies were conducted in Europe and three in Asia. One study focused solely on ARB, five assessed ACEI and ARB separately, and one examined RAS inhibitors as a single group. In terms of control groups, one study included hypertensive patients not exposed to RAS inhibitors, one selected patients with ischemic heart disease (IHD) on other antihypertensive treatments, and one enrolled hypertensive patients on beta-blockers. The degree of adjustment for potential clinical risk factors varied across studies. The Newcastle–Ottawa Scale (NOS) quality scores ranged from 7 to 9, more details are available in Supplementary Tables S1 and S2.

**Table 1:** Characteristics of the included studies

Author, year	Location, setting	Study design/ period	Drug exposure measurement	Comparison	PD outcome assessment	Type of RAS inhibitors(OR, 95%CI)	Number of participants	Adjustment	Quality
Becker et al. [8]	UK, population based	Case–control, 1994–2005	General Practice Research Database	Nonuse of anti-hypertensive drugs	READ Clinical Classification and the OXMIS codes	ACEI 1.08 (0.85–1.37) ARB 0.91 (0.41–2)	Case 3637 Control 3637	Age, sex, general practice, index date, and duration of previous history in the database	8
Ritz et al. [9]	Denmark, population based	Case–control, 2001–2006	National prescription database	Nonuse of ACEI or ARB	Hospital Register	ACEI 1.11 (0.93–1.32) ARB 0.94 (0.74–1.19)	Case 1931 Control 9651	Age, sex, COPD, Charlson index, and other antihypertensive drugs	8
Lee et al. [13]	Taiwan, population based	Cohort, 2005	National Health Insurance Research Database	Patients with hypertension exposed to beta-blockers	ICD-9-CM	ACEI 0.8 (0.64–1) ARB 0.86 (0.69–1.08)	Total 65,001	Time-varying co-morbidities and medications use	8
Warda et al. [10]	Germany, population based	Case–control, 2013–2017	Data Analyzer database	Nonuse of ACEI or ARB	ICD-10	ACEI 0.98 (0.91–1.07) ARB 0.99 (0.88–1.11)	Case 9127 Control 9127	Other antihypertensive drugs, and diagnoses prior to the index data	7
Jo et al. [14]	Korea, population based	Cohort, 2008–2019	Korean Health Insurance Review andAssessment database	Patients with IHD without use of ACEI or ARB	ICD-10	ACEI 0.91 (0.77–1.08) ARB 0.74 (0.65–0.85)	RAS users 31,114 No-user 31,114	Age, sex, follow-up duration, insurance type, comorbid diseases, and concurrent medications	9
Lin et al. [11]	Taiwan, population based	Cohort, 2001–2013	National Health Insurance Research Database	Patients with hypertension without use of ARB	ICD-9	ARB 0.56 (0.51–0.63)	ARB users 49,572 No–user 57,635	Age, sex, diabetes mellitus, stroke, chronic kidney disease, liver cirrhosis, chronic obstructive pulmonary disease, use of statins, and use of each class of antihypertensive drugs	9
Romanowska et al. 2022	Norway, population based	Cohort, 2004–2019	Norwegian Prescription Registry	Nonuse of ACEI or ARB	At least four prescriptions of anti-PD drugs	RAS inhibitors 0.92 (0.89–0.95)	Total 3,22,3672	Sex and education level	8

*ACEI* angiotensin converting enzyme inhibitor; *ARB* angiotensin ii receptor blocker; *COPD* chronic obstructive pulmonary disease; *ICD* international classification of diseases; *IHD* ischemic heart disease; *PD* Parkinson’s disease; *RAS* renin-angiotensin system

### Meta-analysis

#### Use of RAS inhibitors and overall PD risk

**Fig. 2:**
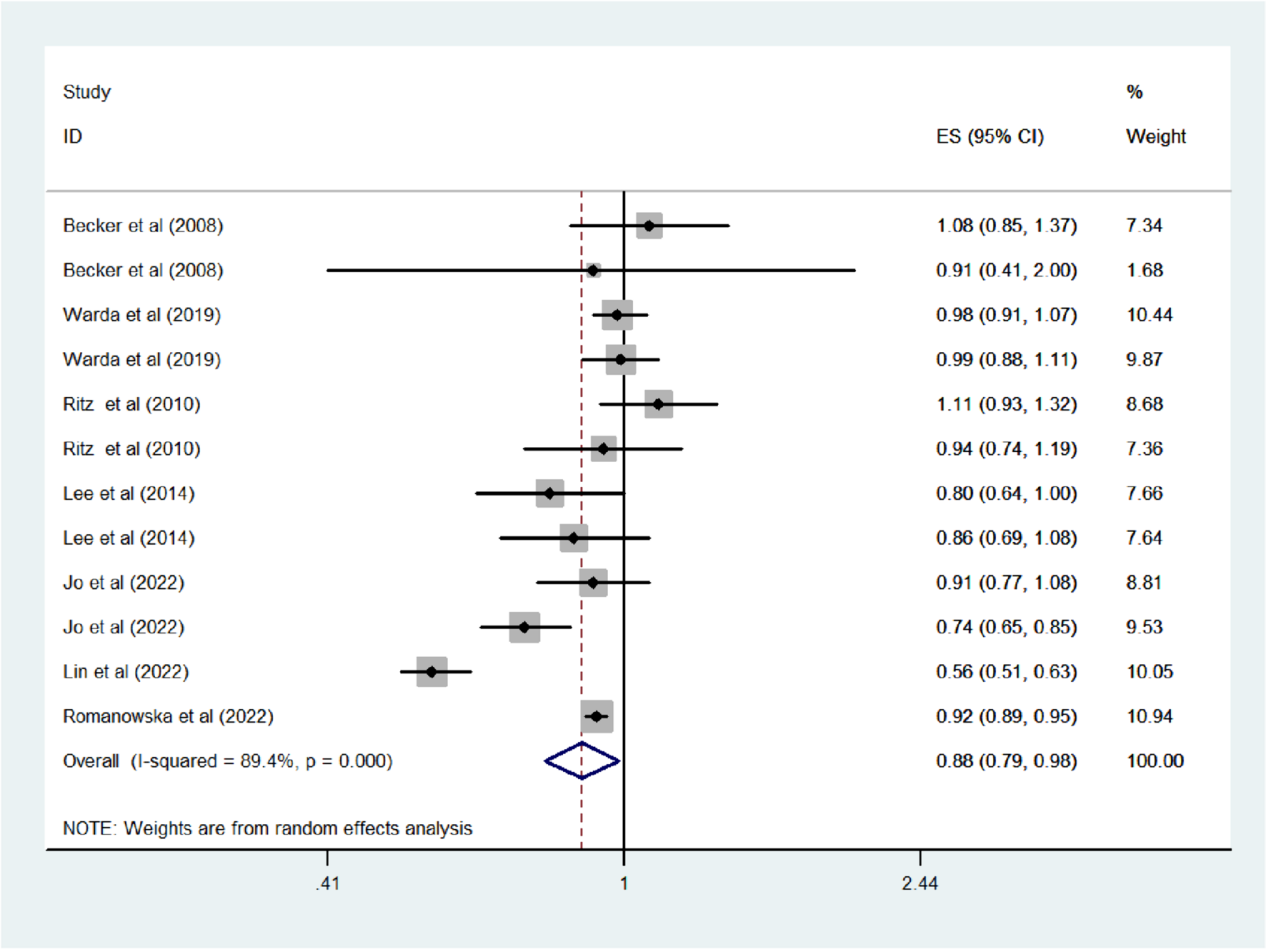
Overall effect estimates for PD and RAS inhibitor use

As shown in Fig. 2, the meta-analysis revealed a significant reduction in the risk of PD with RAS inhibitor use (odds ratio [OR] = 0.88, 95% confidence interval [CI] = 0.79–0.98 *P* = 0.025), although high heterogeneity was found (*I*^*2*^ = 89.4%). No publication bias was detected. Sensitivity analysis by excluding Romanowska et al.’s study, no significant but a trend toward a decrease in PD risk was observed among RAS inhibitor users (OR = 0.88, 95%CI = 0.76–1.02). However, other sensitivity analyses indicated stability in the risk estimates, as no significant change was observed when individual studies were sequentially excluded from the analysis. RAS inhibitors were associated with a reduced risk of PD in cohort studies (OR = 0.79, 95%CI = 0.65–0.96); however, this was not evident in case–control studies (OR = 1, 95%CI = 0.94–1.06). Among the studies accounting for indication bias, RAS inhibitor use was associated with a significantly lower PD risk (OR = 0.76, 95%CI = 0.62–0.92).

#### Associations between different types of RAS inhibitors and PD risk

**Fig. 3:**
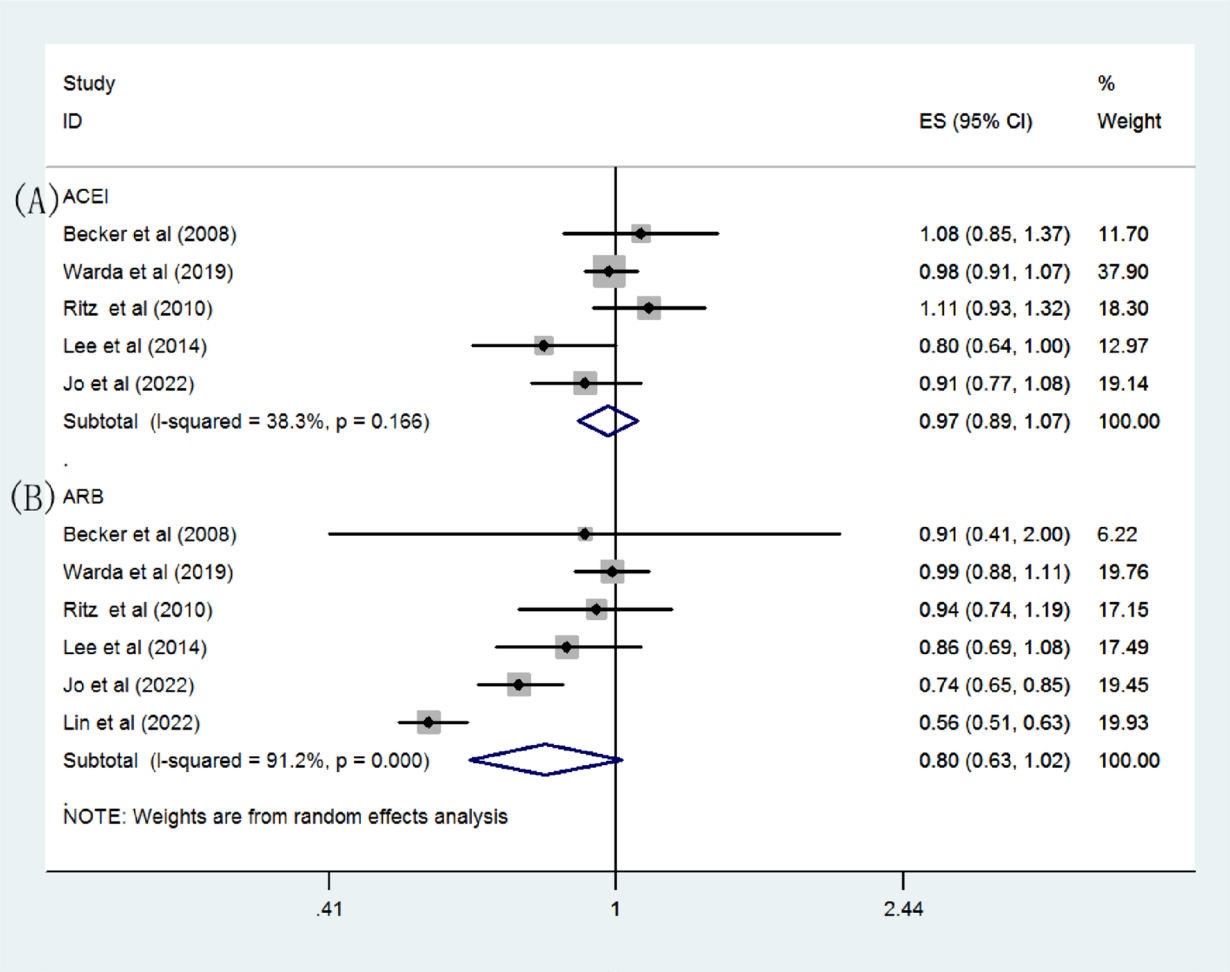
Overall effect estimates for PD and RAS inhibitor use according to subgroups **A** ACEI **B** ARB

Figure 3 presents data from six studies on various RAS inhibitors. Stratification by inhibitor type revealed a pooled OR for PD of 0.97 (95%CI = 0.89–1.07) for ACEI users and 0.8 (95%CI = 0.63–1.02) for ARB users. ACEI use was associated with a decreased risk of PD in cohort studies (OR = 0.87, 95%CI = 0.76–0.99), but this was not replicated in case–control studies (OR = 1.01, 95%CI = 0.94–1.08). Similarly, ARB use was linked with a reduced risk of PD in cohort studies (OR = 0.7, 95%CI = 0.55–0.9), but this was not observed in case–control studies (OR = 0.98, 95%CI = 0.88–1.09).

## Discussion

This study indicates that the use of RAS inhibitors is associated with a lower risk of PD. Furthermore, when considering indications as potential confounders, the findings support a decrease in the risk of PD associated with RAS inhibitor use. Subgroup analyses of cohort studies also showed that both ARB and ACEI use were linked to a reduced risk of PD.

The connection between antihypertensive agents and PD has garnered considerable interest among researchers. Although previous reviews have examined the link between RAS inhibitors and PD, findings have been inconsistent [20, 21]. Mullapudi et al. reported no impact of ACEI or ARB on PD risk in a small-scale meta-analysis [22]. In the most recent systematic review published in 2023, data were not pooled; instead, the findings were descriptive and suggested that RAS inhibitors may serve as prospective therapeutic targets for PD [23]. Our study updates the previous meta-analysis by incorporating four new studies with substantial sample sizes.

Hypertension has been identified as a risk factor for PD in numerous epidemiological studies [24, 25]. Therefore, when examining the relationship between RAS inhibitor use and PD, the reason these drugs were prescribed must be accounted for. Our subgroup analysis by study design revealed discrepancies between case–control and cohort studies. Previous meta-analyses reported no decrease in the risk of PD in patients using ACEI or ARB, which is consistent with the findings of our case–control subgroup analysis [22]. This could be attributed to unmeasured confounders related to drug indications. In three case–control studies [8–10], individuals not taking RAS inhibitors were chosen as the control group, potentially underestimating the protective effect of RAS inhibitors. However, the three newly added cohort studies [11, 13, 14] assessed the association in patients with hypertension or IHD, using patients on other antihypertensive treatments as negative controls. Consequently, our pooled analysis of these cohort studies indicated a lower risk of PD among RAS inhibitor users, suggesting that the lack of confounding by drug indication may explain the negative association reported in earlier meta-analyses. Surprisingly, subgroup analyses based on type of RAS inhibitor showed negative results. Romanowska et al.’s study [12] is relatively new with the largest number of patients among the included studies. In our sensitivity analysis, excluding this study significantly alters the pooled OR; thus, it is speculated that this article has a great impact on the results. However, this study was not included in this subgroup analyses, which may influence the accuracy of findings for ACEI and ARB.

An important consideration is the blood–brain barrier (BBB) permeability of RAS inhibitors. The potential link between RAS inhibitors and PD is substantiated by their pharmacological mechanism. Angiotensin receptors, found in both peripheral tissues and the brain, may alleviate reactive oxygen species (ROS) when inhibited and prevent dopaminergic neuron apoptosis, potentially decreasing PD risk [6, 7]. The effects of RAS inhibitors could be contingent on their ability to penetrate the BBB. Of the studies included in this review, only one [14] assessed the differential impact of BBB-penetrant and non-penetrant RAS inhibitors, finding a decreased PD risk among users of BBB-penetrant ARBs and ACEIs. This is in line with prior research indicating better neuroprotection from BBB-penetrant RAS inhibitors in the context of dementia [26, 27]. Nonetheless, the influence of BBB penetration on PD risk warrants further investigation due to potential limitations in study sample sizes.

The primary strength of this meta-analysis was the inclusion of all published population-based studies to date, providing a comprehensive dataset on the relationship between RAS inhibitor use and PD risk. Additionally, further analyses accounted for the potential confounding effect of drug indications, increasing the precision of PD risk assessment. Finally, the high quality of the included studies reduced bias. However, there were also notable limitations. The most significant limitation was the small number of studies, particularly for subgroup analyses, which could have affected the accuracy of the results. Moreover, high heterogeneity was observed across the studies, likely due to differences in study design, types of RAS inhibitors, and geographical location. Data on the specific types, dosages, and durations of RAS inhibitors were scarce, precluding the identification of exposure parameters related to PD risk. Consequently, further research is necessary to establish if there is a dose–response or duration-response relationship. As with any meta-analysis of observational studies, uncontrolled confounders may have affected the results. Lastly, the potential impact of other antihypertensive drugs [22], such as calcium channel blockers and beta-blockers, was not ascertainable due to insufficient data for a robust subgroup analysis on concomitant drug usage.

In conclusion, this systematic review and meta-analysis suggest an association between RAS inhibitor use and a reduced risk of PD. Both ACEIs and ARBs appear to decrease the risk of PD. Future investigations should consider the classification by inhibitor type, duration and dosage of RAS inhibitors, as well as their BBB penetration properties, to determine their precise effect on PD risk. Comparisons between RAS inhibitors and other antihypertensive medications, in terms of their influence on PD risk, are also warranted.

## Supplementary Information

Below is the link to the electronic supplementary material.

Supplementary file1 (DOCX 17 KB)

## Data Availability

All data were already published in peer-reviewed journals.
