# Supplementary material for: Renin-angiotensin system inhibitor use and risk of Parkinson’s disease: a meta-analysis
Source: Acta Neurol Belg. 2024 Apr 26;125(1):53–60. doi: 10.1007/s13760-024-02560-7 (PMC11876239; doi:10.1007/s13760-024-02560-7)
Supplement: Supplementary file 1 — Supplementary file1 (DOCX 17 KB) [file 13760_2024_2560_MOESM1_ESM.docx]

| **Table S1**  NOS for Assessment of Quality of Included Studies: Cohort Studies | | | | | | | | |
| --- | --- | --- | --- | --- | --- | --- | --- | --- |
| Study | Selection | | | | Comparability | | Outcomes | |
|  | Representativeness of exposed cohort? | Selection of the nonexposed cohort? | Ascertainment of exposure? | Demonstration that outcome of interest was not represent at the start of the study | Comparability of Cohort* | Assessment of outcome | Was follow-up long enough for outcomes to occur | Adequacy of follow up of cohorts |
| Lee et al, 2014 | ★ | ★ | ★ | ★ | ★ | ★ | ★ | ★ |
| Jo et al, 2022 | ★ | ★ | ★ | ★ | ★★ | ★ | ★ | ★ |
| Lin et al, 2022 | ★ | ★ | ★ | ★ | ★★ | ★ | ★ | ★ |
| Romanowska et al, 2022 | ★ | ★ | ★ | ★ | ★ | ★ | ★ | ★ |
| Note: A star denotes a score of 1; * A maximum of 2 stars can be allotted in this category | | | | | | | | |

| **Table S2** NOS for Assessment of Quality of Included Studies: Case-Control Studies | | | | | | | | |
| --- | --- | --- | --- | --- | --- | --- | --- | --- |
| Study | Selection | | | | Comparability | Exposure | | |
|  | Is the case definition adequate | Representativeness of cases | Selection of controls | Definition of controls | Study controls for at least 3 additional factors | Ascertainment of exposure | Same method of ascertainment of exposure | Nonresponse rate |
| Becker et al, 2008 | ★ | **★** | ★ | ★ | ★★ | **★** | ★ | — |
| Ritz et al, 2010 | ★ | **★** | ★ | ★ | ★★ | **★** | ★ | — |
| Warda et al, 2019 | ★ | **★** | ★ | ★ | ★ | **★** | ★ | — |
| Note: A star denotes a score of 1; * A maximum of 2 stars can be allotted in this category | | | | | | | | |
